# Research on the evolutionary history of the morphological structure of cotton seeds: a new perspective based on high-resolution micro-CT technology

**DOI:** 10.3389/fpls.2023.1219476

**Published:** 2023-10-13

**Authors:** Yuankun Li, Guanmin Huang, Xianju Lu, Shenghao Gu, Ying Zhang, Dazhuang Li, Minkun Guo, Yongjiang Zhang, Xinyu Guo

**Affiliations:** ^1^ State Key Laboratory of North China Crop Improvement and Regulation, Key Laboratory of Crop Growth Regulation of Hebei Province, College of Agronomy, Hebei Agricultural University, Baoding, China; ^2^ Beijing Key Laboratory of Digital Plant, Information Technology Research Center, Beijing Academy of Agriculture and Forestry Sciences, Beijing, China

**Keywords:** cotton, seed morphological structure, micro-CT, phenotypic analysis, temporal succession

## Abstract

Cotton (*Gossypium hirsutum* L.) seed morphological structure has a significant impact on the germination, growth and quality formation. However, the wide variation of cotton seed morphology makes it difficult to achieve quantitative analysis using traditional phenotype acquisition methods. In recent years, the application of micro-CT technology has made it possible to analyze the three-dimensional morphological structure of seeds, and has shown technical advantages in accurate identification of seed phenotypes. In this study, we reconstructed the seed morphological structure based on micro-CT technology, deep neural network Unet-3D model, and threshold segmentation methods, extracted 11 basics phenotypes traits, and constructed three new phenotype traits of seed coat specific surface area, seed coat thickness ratio and seed density ratio, using 102 cotton germplasm resources with clear year characteristics. Our results show that there is a significant positive correlation (*P*< 0.001) between the cotton seed size and that of the seed kernel and seed coat volume, with correlation coefficients ranging from 0.51 to 0.92, while the cavity volume has a lower correlation with other phenotype indicators (r<0.37, *P*< 0.001). Comparison of changes in Chinese self-bred varieties showed that seed volume, seed surface area, seed coat volume, cavity volume and seed coat thickness increased by 11.39%, 10.10%, 18.67%, 115.76% and 7.95%, respectively, while seed kernel volume, seed kernel surface area and seed fullness decreased by 7.01%, 0.72% and 16.25%. Combining with the results of cluster analysis, during the hundred-year cultivation history of cotton in China, it showed that the specific surface area of seed structure decreased by 1.27%, the relative thickness of seed coat increased by 8.70%, and the compactness of seed structure increased by 50.17%. Furthermore, the new indicators developed based on micro-CT technology can fully consider the three-dimensional morphological structure and cross-sectional characteristics among the indicators and reflect technical advantages. In this study, we constructed a microscopic phenotype research system for cotton seeds, revealing the morphological changes of cotton seeds with the year in China and providing a theoretical basis for the quantitative analysis and evaluation of seed morphology.

## 1 Introduction

Cotton is the main source of natural fiber and is widely cultivated both in China and worldwide due to its high economic importance (Ruan, 2005). The cotyledon, epicotyl, hypocotyl, radicle and seed coat are the structural components of cotton seed (Maeda et al., 2021). Additionally, cotton exhibits dark-colored pigment spots known as cotton phenol. The seed’s morphological structure is closely associated with its functions, and differences exist in seed functions based on their various morphological structures. The size and shape of seeds are key agronomical traits that influence cotton yield and quality (Wu et al., 2022). Larger and fuller seeds demonstrate superior early growth, uniformity, and higher metrics such as single plant dry matter weight, root-to-shoot ratio, emergence rate, and leaf area (Liu et al., 1997; Wang et al., 2008). Additionally, the size and surface area of seeds can impact their water-holding capacity, rate of moisture absorption, and metabolic rate (Ozarslan, 2002; Feng et al., 2008). Further, there is a positive correlation between kernel-to-coat ratio, and seed oil content (Pahlavani and Abolhasani, 2006). A vibrant seed color, a prominent oil gland, and a full seed are indicative of greater vitality in cotton seed (Wang, 2007). The seed coat is the outermost protective layer of a seed, and its thickness is related to seed germination, drought, and other environmental stresses (Schüler et al., 2014). Knowing the relative thickness of the seed coat can help better comprehend the biological characteristics of seeds (Schüler et al., 2014). A seed cavity is a gas structure that is difficult to measure inside a seed. In a study of the subcutaneous cavity in maize, the subcutaneous cavity volume of maize seed was found to be one of the most significant factors affecting the grain breakage rate (Hou et al., 2019). However, there is no clear research on the formation and quantification of cotton seed cavities. Therefore, accurate measurement of seed morphological structures is of great significance for exploring seed functions and environmental adaptability. However, cotton seeds display irregular morphology. Traditional measurement methods, like using calipers to measure the linear dimensions of seeds or dissecting seeds to obtain internal physical parameters, produce unsatisfactory outcomes. Consequently, this impedes the progress of studying the morphological structure of cotton seeds. Therefore, agronomists urgently need accurate seed structure analysis methods to study the functional morphological relationships of seeds.

With the continuous advancement of agricultural digital technology and imaging technology, notable progress has been made in the analysis and evaluation of seed morphological structures. Regarding the methods used now for seed imaging, there are mainly four key aspects. The first part involves using two-dimensional (2D) images to examine seed shape. Capturing 2D images allows for the extraction of valuable information regarding the external morphology of seeds, including their size, shape, color and texture (Hacisalihoglu and Armstrong, 2023). 2D images commonly comprise a range of image types, such as red-green-blue (RGB) images, thermal imaging, and fluorescence images. Zhong et al. (2016) extracted the thickness index of wheat seeds using two-dimensional image light projection. In a study by Brodersen and Kuhl (2023) focusing on seaweed seeds, it was observed that photosynthesis in the seed sheath enhances the availability of oxygen in the central region of the seed under light exposure. This, in turn, increases respiratory energy production for biological synthesis and relieves internal oxygen deprivation within the seed. Additionally, Kranner et al. (2010) identified a significant correlation between temperature distribution changes and the seed imbibition process. Thermal imaging technology has demonstrated its viability as an alternative to conventional methods for assessing seed vigor. However, many agronomic traits need to be analyzed in a three-dimensional model (3D).

The second part focuses on the detection of the chemical composition of seeds using spectral technology. Spectral imaging is a fusion of spectroscopy and imaging technology. This method utilizes the absorption, scattering, or transmission characteristics of seeds to acquire chemical composition information within the seeds across varying wavelengths of light. This method offers several advantages, including high speed, high efficiency, non-contact, non-destructive, and reliable results. Commonly utilized hyperspectral images, multispectral images, near-infrared (NIR) imaging, and so on. In the current study, Fourier transform near-infrared (FT-NIR), dispersive diode array (DA-NIR) and hyperspectral imaging (HSI) have been successfully used to detect the quality of seed components such as protein, oil, water and starch. Furthermore, these methods have demonstrated the capability to predict chemical quality traits (Hacisalihoglu and Armstrong, 2023). Moreover, although fluorescence and thermal infrared images are not composed of a continuous spectrum of visible light wavelengths, they still involve the interaction between matter and light as well as the process of energy conversion of light. Therefore, they can also be classified as part of the spectrum. The combination of fluorescence and hyperspectral imaging also holds great potential for assessing heavy metal content (Zhou et al., 2023). Additionally, the fusion of proximal spectral phenotyping and 3D modeling registration has emerged as a new development trend (Liu et al., 2020), but it has received limited attention in the field of seed research.

The third component is the 3D imaging technology using serial sections. Serial sections entail physically slicing the sample into thin slices. Next, each slice is observed and imaged under a microscope before layering the images to reconstruct the 3D structure of the entire sample. For instance, Ogawa et al. (2000) reconstructed the 3D morphological structure of rice seeds using consecutive sectional images. Commonly used 3D imaging techniques that make use of serial sections include traditional transmission electron microscopy (TEM) and scanning electron microscopy (SEM). This method provides detailed internal ultrastructural features of seeds, such as cell morphology and number (Rashid et al., 2020). In a study examining protein and vacuole formation in pea seed cotyledons, researchers reconstructed serial sections of tissues obtained on the 12th and 15th days, providing evidence for the possible formation of protein bodies (Craig et al., 1979). In a distinct study, Bhawana et al. (2014) utilized focused ion beam-scanning electron microscopy (FIB-SEM) to capture the 3D features of aleurone cells in Arabidopsis seeds. Consecutively slicing experimental samples is a time-consuming and destructive process that can lead to the exclusion of specific regions of interest (Prior et al., 1999). Moreover, incorrect thickness intervals between slices can cause deformation in the sections (Yamane et al., 2022).

The final part is 3D imaging technology. Commonly used 3D imaging techniques in seed research comprise magnetic resonance imaging (MRI), X-ray micro-computed tomography (Micro-CT, also known as μCT), confocal microscopy, and structured light imaging technology. 3D imaging technologies have the capability to examine anatomical internal structures without the need to slice them (Mohoric et al., 2009). Micro-CT utilizes X-rays for scanning and reconstructing the 3D structure of samples. This technique can provide high-resolution images, making it valuable for analyzing the internal structure of seeds (Yu et al., 2022). MRI employs magnetic fields and radio waves to generate high-contrast images containing anatomical information. MRI can offer insights into the internal structure and water distribution of seeds in seed research (Metzner et al., 2015). A 3D microscope employs lasers or near-infrared light sources for scanning samples and generating 3D models of seeds using stacked images. Structured light imaging involves using structured patterns generated by light sources to obtain the 3D external shape of samples. Despite having relatively larger errors compared to other techniques (Yu et al., 2022), structured light imaging still has certain applicability in studying the external morphology of seeds. In principle, MRI signals are based on the flow of water, while X-ray tomography is based on the differential absorption of X-rays by the sample, but compared with MRI, micro-CT can achieve higher spatial resolution (Metzner et al., 2015). Laser confocal microscopy (LSM) can nondestructively observe and analyze the internal microstructure of cells by constructing a 3D model of samples through 3D layer imaging, but it is slower than micro-CT imaging (Gubatz et al., 2007; Fanuel et al., 2018). So it seems appropriate to use micro-CT methods to explore seed structures.

In recent years, the application of micro-CT technology has become increasingly prevalent in the analysis of crop seeds. This innovative technology has been used to analyze the 3D morphological structure of seed organs in crops like rice, wheat, sorghum and maize. For example, micro-CT technology was used to measure the chalkiness index and quantify the crack size of rice seeds, promoting the genetic analysis of rice chalkiness regulation and quality evaluation in production (Su and Xiao, 2020). Le et al. (2019) assessed the morphology of wheat grain and its different compartments, quantifying the crease shape for each grain. Crozier et al. (2022) extracted phenotypic quantitative data from sorghum grain, including embryo volume, endosperm hardness, endosperm texture, endosperm volume, pericarp volume, and seed kernel volume, to enable the identification of genotypes with superior structural characteristics. Additionally, Yin et al. (2021) used micro-CT technology to analyze the variation of maize kernels from base to top, revealing the positional effect on grain growth and development. Overall, micro-CT technology offers a unique 3D perspective on characterizing seed morphological structure and vast potential for exploring the relationship between seed morphological structure, quality, and water transport (Warning et al., 2014). This technology represents a robust tool for studying the relationship between seed morphological structure and function and exploring the mechanisms of seed quality control. However, although this technology has been widely used on various crops, the micro-CT-based analysis technology of cotton seed has not yet been established, and the 3D structural phenotypic information on cotton seed remains undefined.

Over time, different breeding goals and specific production issues have resulted in varying growth characteristics and ecological adaptability of plant varieties, which can be reflected in significant differences in crop phenotypes. Seed phenotype plays a crucial role in contributing to crop phenotypes and reflects the profound genetic changes caused by intentionally or unintentionally human selection. For example, significant changes have occurred in the morphology and size of wheat grains during domestication and breeding due to the demand for flour protein particle content and hydrolytic enzyme activity (Gegas et al., 2010). This change has led to the classification of wheat cultivars, such as common wheat, hard wheat, cone wheat, and dense wheat. The shape of grains is divided into four categories: angular, oval, cylindrical, and elliptical, based on the shape of wheat seed kernels. Production-wise, wheat is often divided into two types based on the color of the grain: red skin and white skin. The red-skinned variety has a thicker skin, poor ventilation, and long dormancy, but is resistant to grain sprouting (Lang et al., 2021). However, seed morphological structure evolution is often ignored, and more focus is given to plant architecture evolution because of the similarity between germplasm. Cotton seeds are mainly identified based on features such as color and the chemical composition content of the seed kernel, as there is no clarity on the evolutionary characteristics of the seed morphological structure of cotton.

Given the absence of a 3D structure in cotton seeds and the challenge of accurately quantifying the internal structure, along with the unclear understanding of seed evolution, this study collected representative seed samples from different years and employed micro-CT technology to analyze the morphological structure of the seeds. The results elucidated the process and trend of changes that occurred in the cotton seed throughout the different years. It was hypothesized that (1) the parameters of the micro-CT equipment can resolve the 3D morphological structure of the cotton seed; (2) there is a significant correlation around the morphological indicators of the cotton seed; and (3) the morphology of the cotton seed has distinct temporal characteristics as breeding progresses. The purpose of this study is to establish a micro-CT-based method for obtaining the morphology of the cotton seed, analyze the internal morphological characteristics of cotton, and investigate the evolution of cotton seed morphological structure. This study provides a high-throughput method for assessing the morphology of the seed morphological structure and a theoretical basis for the quantitative evaluation and analysis of the seed morphological structure.

## 2 Materials and methods

### 2.1 Experimental materials

The experiment was conducted at the Digital Plant Laboratory of the Beijing Academy of Agriculture and Forestry Sciences in 2022. The study incorporated 102 diverse types of cotton seeds (**Table S1**). 83 of these seeds were cotton varieties with explicitly identifiable breeding years and were grouped into four years based on the breeding timeline (Yu, 2018). Years 1-4 pertain to cotton varieties that were introduced from overseas or domestically cultivated and widely promoted in China within the years 1904-1958, 1958-1970, 1970-1990, and 1990-2020, respectively.

Among them, 51 seeds and 5 seeds were sown and harvested in Hebei Province and Xinjiang Province, respectively, in 2020. Sixty-one samples were stored in the national cotton germplasm resources Mid-Term Genebank at a temperature of 0°C ± 2°C and a relative humidity of 50% ± 7%.

### 2.2 Cotton seeds retrieving and analyzing methods establishing

#### 2.2.1 Micro-CT image acquisition

The micro-CT device used for this study was the SkyScan 1172 (Bruker, USA). The scanning resolution was set at 2K (2000×1332), with a scan pixel pitch of 12.86 µm and an angular step of 0.400°. The scan was conducted using a voltage of 40 kV and a current of 250 µA, and each individual scan required approximately 29-30 minutes to complete. To improve the accuracy and efficiency of scanning, three cotton seeds were secured on a homogeneous foam panel, and the panel was fixed to the rotary table of the micro-CT scanning equipment to decrease the influence of other fixed matrices on image grayscale. After each scan, 488 images (. TIF) were acquired for each variety, resulting in a total size of 2.41 GB. The CT Scan NRecon software (Bruker, US) was utilized for image reconstruction. Each reconstructed image had a format of BMP with a resolution of 2000×2000px, and their sizes ranged from approximately 3 to 4 GB.

#### 2.2.2 Single seed segmentation

First, the original image was downsampled by a factor of 3. The threshold for image binarization was automatically determined using the Otsu algorithm (Otsu, 1979). Morphological operations were employed to eliminate internal holes and generate an image mask. Next, the three-dimensional watershed algorithm (Neubert and Protzei, 2014) was utilized to segment individual seed kernels. To extract micro-CT images of individual seed kernels, the segmented image was combined with the original image using the “AND” operation.

#### 2.2.3 Seed kernel phenotyping pipeline

**Figure 1:**
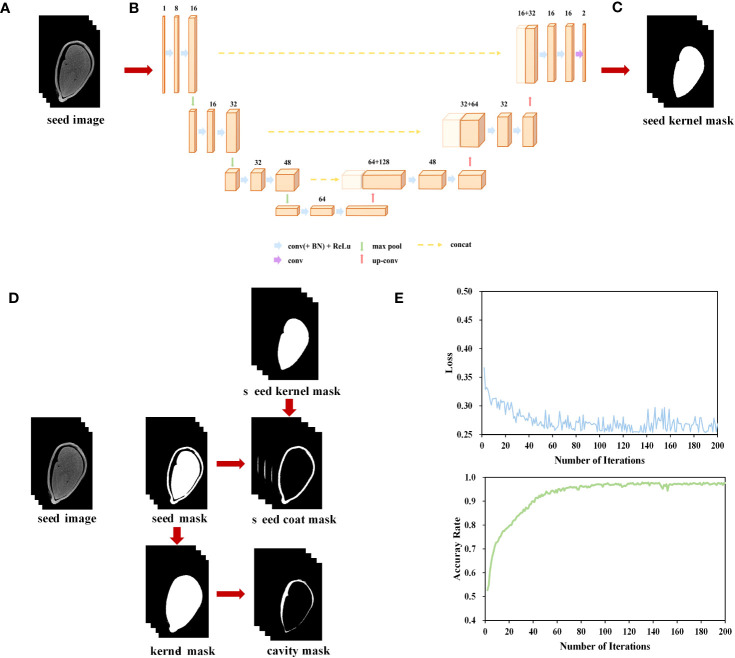
Micro-CT-based cotton seed imaging processing flow. **(A)** micro-CT image sequence. **(B)** U-net network. **(C)** Segment seed kernel mask. **(D)** Segment seed coat and cavity. **(E)** Loss and accuracy curves on training set.

In the micro-CT images of cotton seeds, there is high pixel grayscale in their seed kernels and seed coats. To enhance the efficiency of seed image processing, we aim to develop a phenotyping pipeline. The dataset we used comprises 286 scanned images of 20 seed kernels. Next, we employed the effective interactive segmentation (EISeg) method described by Hao et al. (2022) to manually label the embryonic region of these images (**Figure 1A**). Among these structures, the seed kernel is more easily identifiable and labelable. However, manually labeling the seed coat and cavity structure resulted in significant errors. Therefore, we only labelled the kernel for model training. Additionally, the data was divided into training and testing sets with a ratio of 8:2, chosen randomly.

We utilized the PyTorch - cpu0.8.2 deep learning framework, along with libraries such as SimpleITK, numpy, scipy, skimage, and vedo in Python (3.9.0). The U-net 3D network architecture was illustrated in **Figure 1B**. This network consisted of an encoder and a decoder, taking in images of size 32× 128× 128 and producing seed kernel masks of size 32× 128× 128. The network was divided into 4 layers, performing 5 down sampling operations during the encoding phase and 5 up sampling operations during the decoding phase. Each layer was composed of two Conv3D-BN-ReLU modules connected by residual connections. The number of convolutional kernels in each layer was 16, 32, 48, 64, and 96, respectively. MaxPool and ConvTranspose were used for downsampling and upsampling, respectively. LeakyReLU was employed as the activation function in the activation function and normalization layer, while InstanceNorm was used as the normalization layer to enhance the model’s expressive power. To restore the low-resolution feature maps to the original image resolution, bilinear interpolation was applied in the upsampling layer. Furthermore, the model’s output was normalized and probabilized using the Softmax and Sigmoid functions. The model configuration included setting the Epoch to 300, the learning rate to 0.0001, the Batch_size to 4, and performing the training using the Adaptive Momentum Estimation (Adam) optimization algorithm. The loss function used was Dice_Loss. The accuracy of the network was evaluated using the Dice similarity coefficient (DICE) and Intersection over Union (IoU).

During both the training and inference stages of the model, the input images were processed in a chunking manner. In the training stage, 32× 128× 128 volumetric data samples were randomly extracted from the original images and their corresponding labels as inputs to the network (**Figure 1B**). During the inference stage, volumetric data of size 32 ×128 ×128 was sequentially extracted with a stride of (24, 96, 96), and then fed into the segmentation network for inference, ultimately obtaining the seed kernel mask image (**Figure 1C**).

Throughout 200 training iterations with a learning rate of 0.0001, the loss consistently decreased, converging to a model’s accuracy of 97.7% (**Figure 1E**).

#### 2.2.4 Seed coat and cavity extraction

We utilized the Otsu algorithm (Otsu, 1979), a basic image processing technique, to segment the seed coat and cavity based on the disparity in gray levels between the target object and the background in the given image. We performed a gray-level histogram analysis on each individual seed kernel to determine the threshold for binarization and obtain the seed mask image. Subsequently, this image was utilized to create a mask encompassing the contour of the seed. Finally, by utilizing the “AND” operation, the masks corresponding to the seed coat and cavity were merged with the original image, resulting in the production of seed coat and cavity images (**Figure 1D**).

Open-source medical imaging processing tools, including Itk-SNAP (Yushkevich et al., 2006) and 3D Slicer (www.slicer.org) (Fedorov et al., 2012), were employed for rendering and 3D visualization purposes.

### 2.3 Sampling and measurements

#### 2.3.1 Micro-CT analysis

Based on the target mask image and the target surface model reconstructed by the Marching Cube algorithm (Lorensen and Cline, 1987). Based on the surface model, 11 phenotypic traits of cotton seeds, comprising seed, seed coat, seed kernel, and cavity morphological structure (**Table S2**) were extracted. It is important to mention that the seed surface area is also known as the seed coat surface area, and the usage of these terms in the paper depends on the context of the paragraph.

#### 2.3.2 Manual measurement

In order to compare the measured dimensions of seeds with their corresponding extracted data, it is necessary to use a vernier caliper to measure and record their length, width, and thickness. We defined length as the maximum dimension of the seed, while width is the maximum dimension perpendicular to its length (Hu et al., 2018). We defined the thickness as the straight-line size perpendicular to both the length and width directions (Hu et al., 2018).

### 2.4 Seed morphological structure evaluation indicators

The seed coat specific surface area is expressed by the ratio of the seed coat surface area to its volume, which can reflect the surface area of the seed coat per unit volume. The larger the specific surface area of the seed, the greater its exposure to the surrounding environment.


(1) $$\begin{array}{c} \text{Seed coat specific surface area}\;\left(m^{2}/m^{3}\right)=\text{Seed coat surface area}(m^{2})/\text{Seed coat volume}(m^{3}) \end{array}$$

The seed coat thickness ratio is defined as the average seed coat thickness divided by the seed thickness. The seed coat thickness ratio represents the relationship between the thickness of the seed coat and that of the seed, with a higher value indicating thicker seed coat.


(2) $$\begin{array}{c} \text{Seed coat thickness ratio}=\text{Average seed coat thickness }(\text{m})/\text{Seed thickness }(\text{m}) \end{array}$$

The seed density ratio is defined as the ratio of the cavity volume of to the seed kernel volume. It is commonly used to evaluate the internal morphological structure of seeds, with a smaller SDR value indicating a denser internal structure of the seeds.


(3) $$\begin{array}{c} \text{Seed density ratio}=\text{Cavity volume}(m^{3})/\text{Kernel volume}(m^{3}) \end{array}$$

### 2.5 Data analysis

Experimental data were organized using Microsoft Excel 365 (Microsoft Corporation, USA) with statistical analyses carried out using SPSS Statistics 25 (IBM Corporation, USA) for variance analysis and variety clustering. ANOVA analysis of variance (generalized linear model) was used. When the data conformed to a normal distribution, we used LSD multiple comparisons for data that conforms to normality. For data that did not conform to normality, in order to avoid false positive results, we used Bonferroni multiple comparison results. The least significant difference (LSD) method was applied for multiple comparisons, with significant differences between different seed parameters compared on a *P*<0.05 level. The average seed coat thickness indicator was excluded from the clustering analysis due to its insignificant change. Therefore, ten seed phenotypic indicators were standardized using the Z-score method and classified using the Ward method in combination with squared Euclidean distance as the similarity measure, to categorize the indicators of different cotton varieties.

## 3 Results

### 3.1 Establishment of micro-CT acquisition and analysis method for cotton seeds

**Figure 2:**
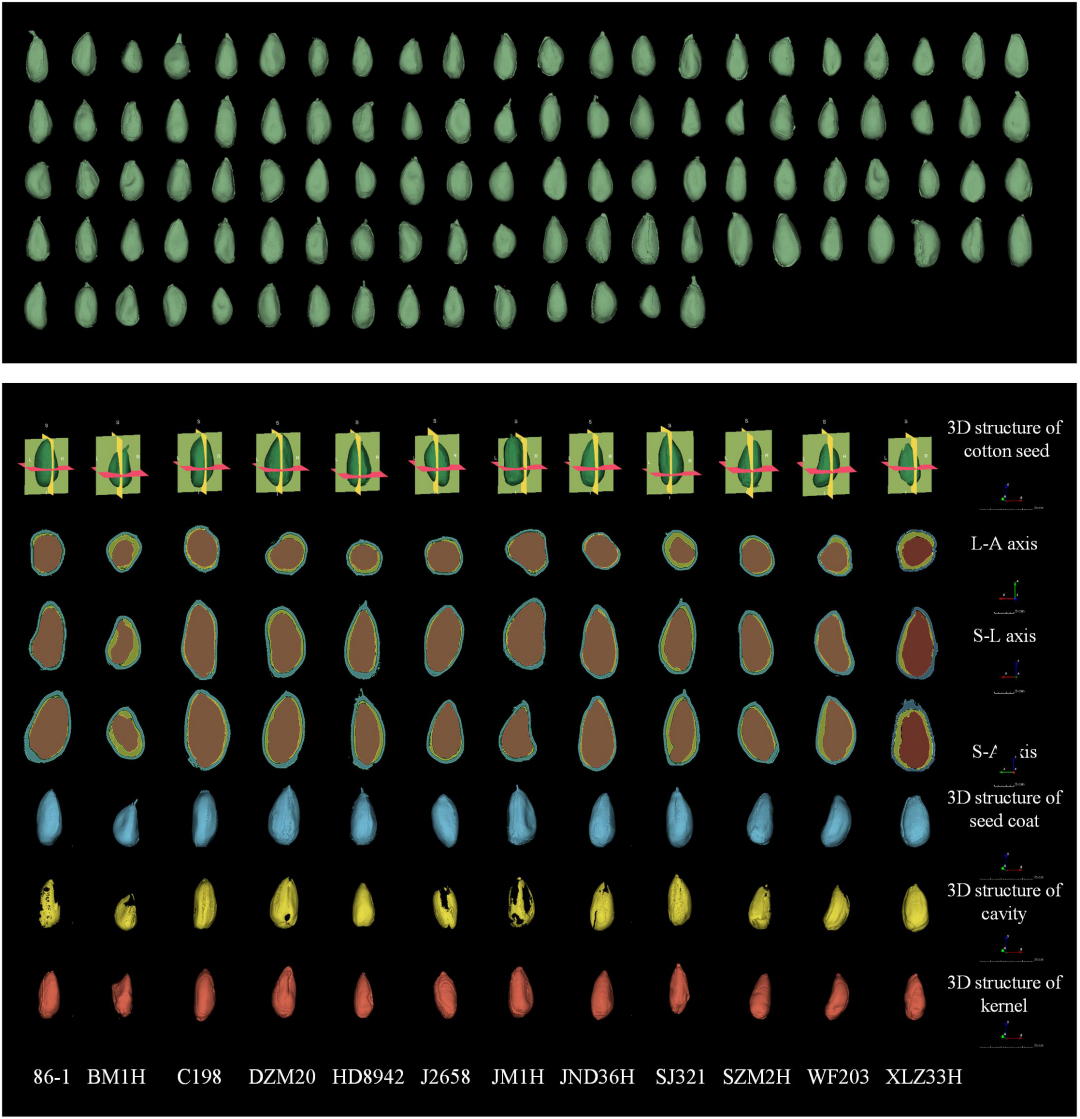
Three-dimensional reconstruction of 102 cotton seed (green) and three-dimensional reconstruction images and three-dimensional view of seed coat (blue), cavity (yellow), and seed kernel (red) of 12 seeds. The seeds shown in the figure are represented using the RAS coordinate system and demonstrate consistent orientation, measured in mm. The three-dimensional morphological structure was scaled to 25 cm, and the image scale for the three orthographic views was set to 5 cm.

Since cotton is a dicotyledonous crop, the cotyledons of cotton seeds are curled and closely connected to the radicle and hypocotyls. Meanwhile, distinguishing between the radicle, hypocotyl, primordial epicotyl, and two cotyledons was difficult in our micro-CT images. Thus, these anatomical components were regarded as a unified morphological structure known as the seed kernel for the purpose of segmentation and computational analysis. **Figure 2** presented 3D reconstruction images of the 102 cotton varieties and the seed coat, cavity, and seed kernel of 12 cotton varieties from various angles.

**Figure 3:**
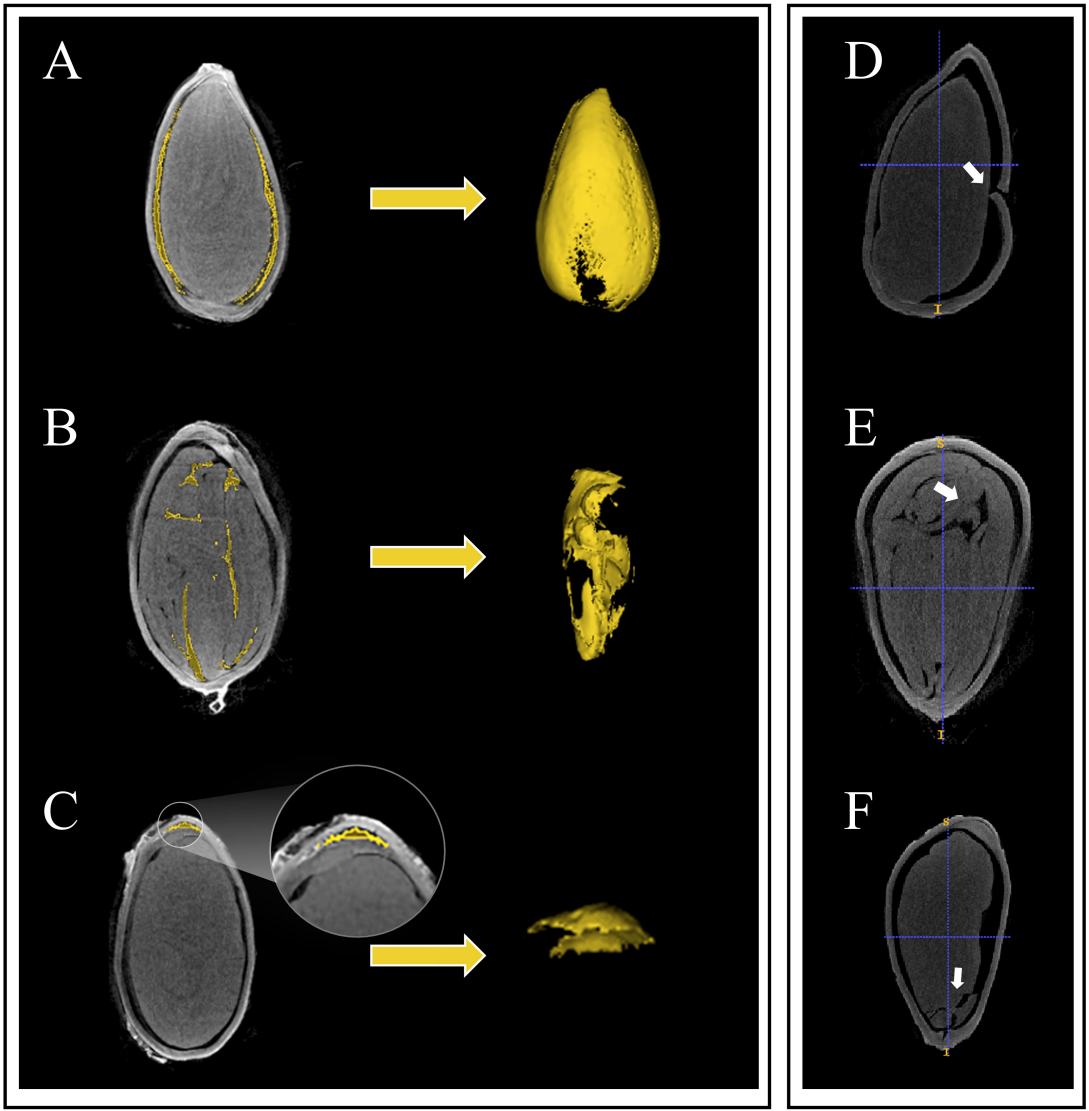
Three types of the inner structures and seed damage in cotton seeds are depicted. Among them, **(A)** represents the cavity between the seed coat and the kernel, **(B)** depicts the cavity inside the seed coat and endosperm remnants, **(C)** shows the cavity inside the seed kernel, **(D)** represents damage to the seed coat, **(E)** shows the breach between seed kernels, and **(F)** represents damage to the seed kernel.

Upon observing the micro-CT scan images of cotton seeds, it became evident that the cavity structure occurs between the seed kernel and seed coat (**Figure 3A**). Three other types of cavity morphological structures also existed, namely the internal cavity of the seed kernel (**Figure 3B**), the cavity between the endosperm residue and the seed coat (**Figure 3C**), and the cavity between the internal and external seed coat. However, unlike other crop seeds, the latter three cavity morphological structures of cotton seeds are atypical and mainly occur in dry, dehydrated, or even dead seeds. Thus, this article solely focuses on the cavity between the seed kernel and seed coat.

### 3.2 Analysis of phenotypic indicators of cotton seeds

**Figure 4:**
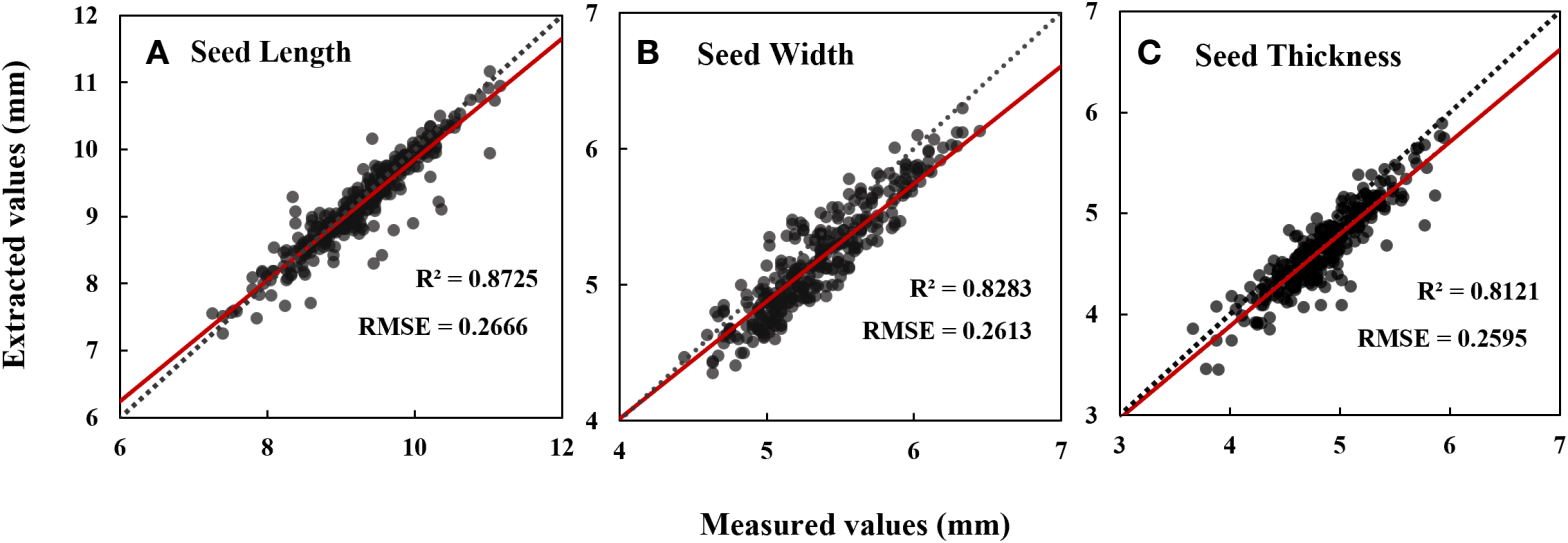
Data evaluation of seed length **(A)**, seed width **(B)** and seed thickness **(C)** measured values extracted based on CT images. N=306. Date represents mean ± SE (3 biological replicates, n=9, 15 and 26 plants, respectively), letters above the bars indicate significant differences at the level of P<0.05.

Eleven phenotypic traits of cotton seeds were obtained through the analysis of micro-CT images. Descriptive statistics showed that the average seed length of 102 cotton seeds was 9.22 mm, with a relatively small standard deviation and coefficient of variation values (**Table 1**). The average values for seed width and thickness were also stable. The thinnest and thickest seed coats had average thicknesses of 0.10 mm and 0.21 mm, respectively, but relatively larger coefficient of variation values than seed length, width, and thickness. Kernel volume and kernel surface area had larger coefficient of variation values, with values of 0.21 and 0.14, respectively (**Table 1**). On average, the seed kernel accounted for 54% of the total seed volume, with the remaining 46% comprising the seed coat and internal cavity. Notably, the coefficient of variation of the cavity was as high as 0.71 (**Table 1**). Comparing the data obtained through micro-CT with those obtained through manual measurements, the coefficients of determination for seed length, width, and thickness were 0.87, 0.83, and 0.81, respectively (**Figure 4**).

**Table 1:** Descriptive statistics for the phenotypic indicators of 102 cotton seeds.

Morphological trait	Unit	Mean ± SD ^a^	CV ^b^	Range
Seed Length	mm	9.22 ± 0.70	0.08	7.26-11.16
Seed Width	mm	5.40 ± 0.50	0.09	4.44-8.49
Seed Thickness	mm	4.86 ± 0.41	0.08	3.67-6.91
Seed Volume	mm³	100.34 ± 15.40	0.15	63.3-150.71
Seed Surface Area	mm²	148.51 ± 21.14	0.14	106.52-223.83
Seed Kernel Volume	mm³	54.25 ± 11.59	0.21	26.82-95.37
Kernel Surface Area	mm²	89.26 ± 12.51	0.14	61.08-129.92
Seed Cavity Volume	mm³	10.33 ± 7.31	0.71	0.09-41.94
Seed Coat Volume	mm³	23.99 ± 4.27	0.18	12.96-41.27
Average Seed Coat Thickness	mm	0.16 ± 0.02	0.12	0.1-0.21
Seed Fullness	%	0.54 ± 0.06	0.12	0.3-0.67

^a^ SD is the abbreviation for standard deviation; ^b^ CV is the abbreviation for Coefficient of variation.

**Figure 5:**
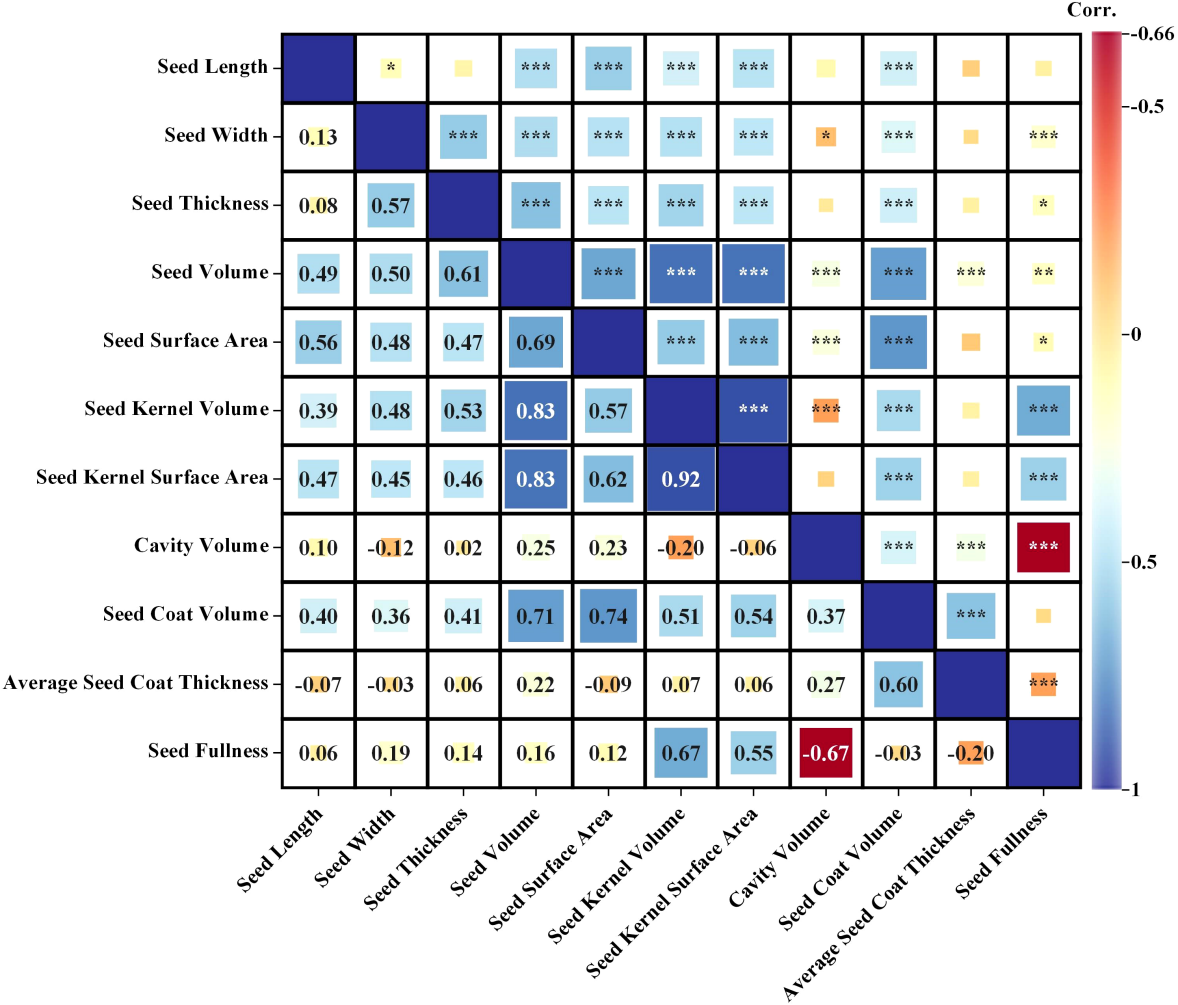
Correlation analysis of 11 phenotypic indicators (Seed Length, Seed Width, Seed Thickness, Seed Volume, Seed Surface Area, Kernel Volume, Kernel Surface Area, Seed Coat Volume, Seed Cavity Volume, Average Seed Coat Thickness and Seed Fullness). Significance *< 0.05, **< 0.01; ***< 0.001.

Through Pearson correlation analysis of 11 phenotypic characteristics of cotton seeds (**Figure 5**), it revealed a highly significant positive correlation between the volume and surface area of the seed and the volume and surface area of the seed kernel, as well as the volume of the seed coat (r = 0.57~0.83, *P*< 0.001). There was a weak correlation between the cavity volume and the seed coat volume (r = 0.37, *P*< 0.001), and a weak correlation with other phenotypic characteristics (r < 0.30, *P* < 0.001) (**Figure 5**). Additionally, the volume and surface area of the seed kernel showed a positive correlation with the volume and surface area of the seed and with the volume of the seed coat (r = 0.51~0.83, *P*< 0.001) (**Figure 5**). In addition, seed coat volume was positively correlated with the volume and surface area indexes of other phenotypic features (r = 0.51~0.74, *P*<0.001) (**Figure 5**). However, the seed thickness was moderately and positively associated with the seed coat volume (r = 0.60, P < 0.001), and had a weak correlation with the seed volume and cavity (r< 0.03, P< 0.001), and was not significantly correlated with other phenotypic characteristics (**Figure 5**).

### 3.3 Changes trends in cotton seed morphological structure in different years

Comparisons of seed phenotypic characteristics from different years (**Figure S1**), from 1904-1958 to 1958-1970, showed significant changes in length, width, seed volume, seed kernel volume, seed surface area, seed coat volume, cavity volume, and seed fullness. Conversely, the average seed coat thickness had a minimal and insignificant decrease (**Figure S1**). 1958-1970 had a significantly smaller length (8.56 mm), thickness (4.71 mm), seed volume (88.12 mm^3^), seed kernel volume (55.28 mm^3^), and seed coat volume (20.19 mm^3^) than the other years (**Figure S1**). From 1958-1970 to 1970-1990, all indicators, except for seed fullness, showed an upward trend, and most indicators had significant differences compared to 1958-1970 (**Figure S1**). However, when comparing the indicators of 1904-1958, 1958-1970, and 1970-1990, 1904-1958 and 1970-1990 had less significant differences in the various indicators (**Figure S1**). In 1990-2020, the degree of decline in length, width, thickness, seed surface area, seed coat volume, average seed coat thickness, and seed fullness compared to 1970-1990 was not very significant (**Figure S1**). Nevertheless, there was a remarkable decrease in seed volume, seed kernel volume, and seed kernel surface area, by 8.56%, 14.11%, and 7.82%, respectively (**Figure S1**).

**Figure 6:**
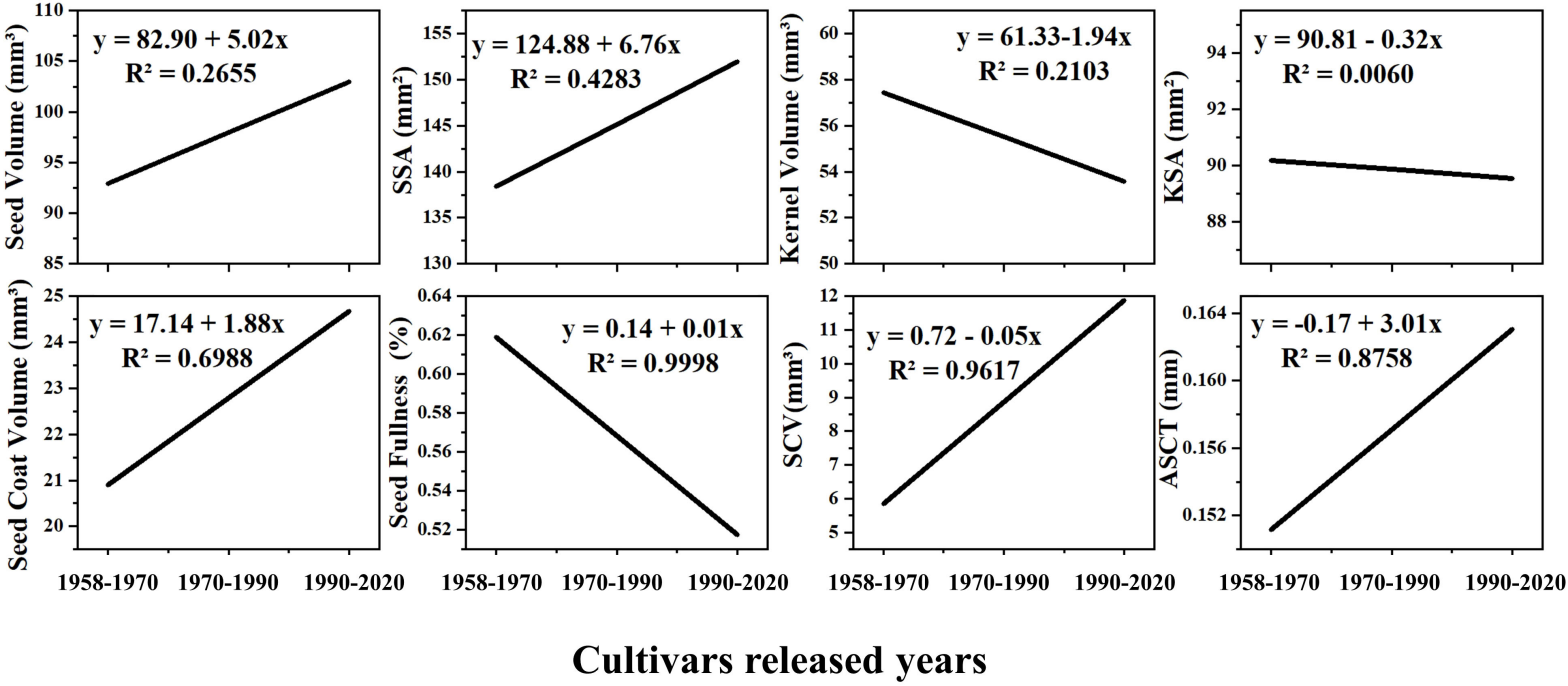
The trend of seed morphological structure change of cotton varieties independently cultivated in China. SSA, Seed Surface Area; KSA, Seed Kernel Surface Area; SCV, Seed Cavity Volume; ASCT, Average Seed Coat Thickness. The cultivars released years involve cotton varieties cultivated from the country in 1958-1970, 1970-1990 and 1990-2020, respectively.

Comparing the changes in the seed morphological structure of varieties domestically bred in China from 1958 to 2020 (**Figure 6**), we observed an upward trend in seed volume and surface area, seed coat volume, cavity volume and average seed coat thickness of domestically bred varieties. They increased by 11.39%, 10.10%, 18.65%, 115.76%, and 7.85%, respectively (**Figure 6**). However, the seed kernel volume, seed kernel surface area, and seed fullness showed a downward trend, decreasing by 7.01%, 0.72%, and 16.25%, respectively (**Figure 6**). Among them, the seed coat volume and average seed coat thickness had goodness-of-fit values of 0.70 and 0.88, respectively (**Figure 6**). The cavity volume had an R² value of 0.9671, and the goodness-of-fit of seed fullness was the highest, approaching 1, indicating high predictability (**Figure 6**).

### 3.4 Similarity and classification of cotton seeds from different years

**Figure 7:**
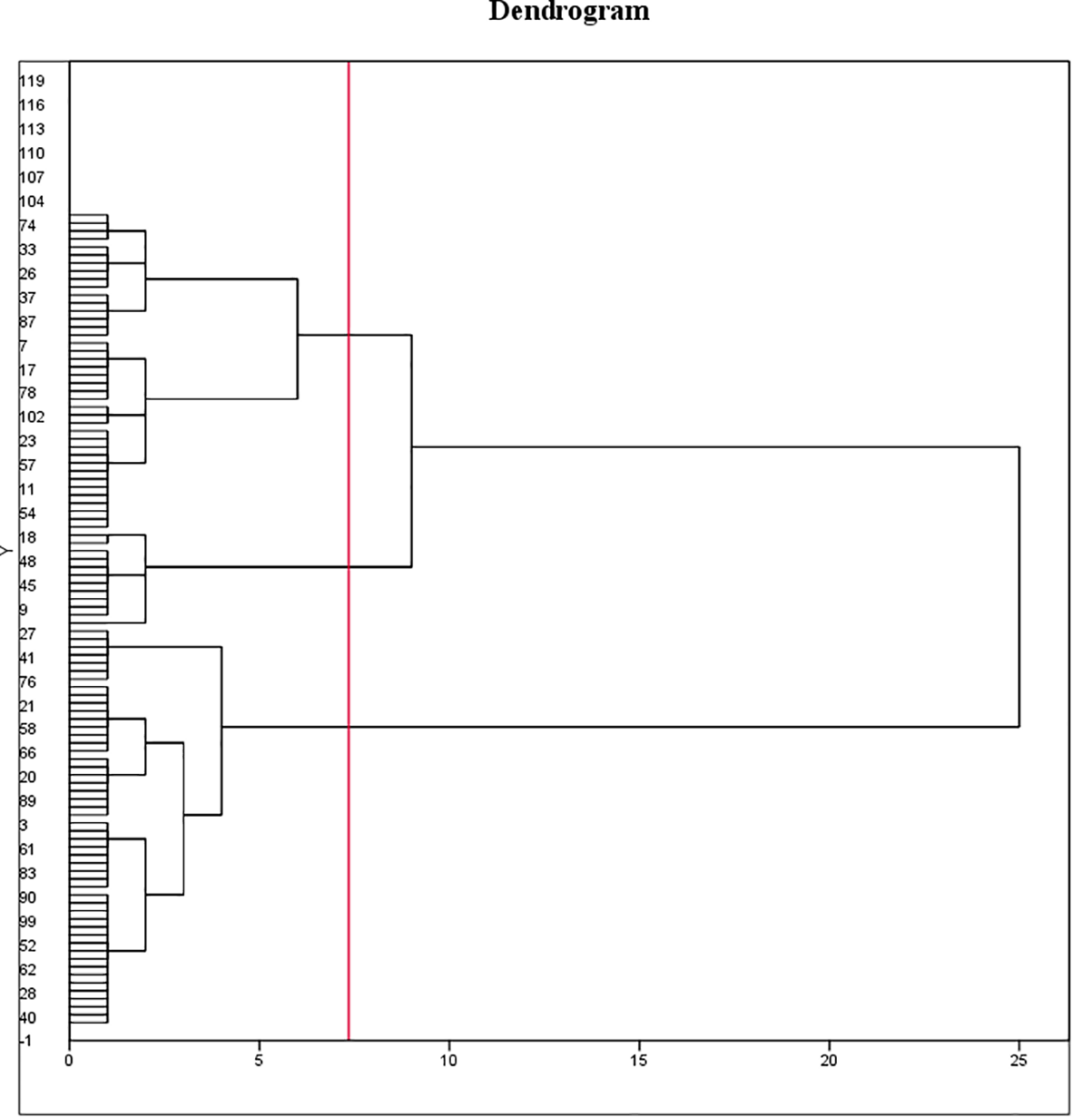
Clustering of 102 cotton varieties. Ten seed phenotypic indicators were standardized using the Z-score method and classified using the Ward method in combination with squared Euclidean distance as the similarity measure, to categorize the indicators of different cotton varieties. The 102 varieties are divided into three categories. The first cluster comprised 46 varieties, accounting for 45.1% of the total varieties. Of these, one variety was from 1904-1958, three varieties from 1958-1970, four varieties from 1970-1990, 32 varieties from 1990-2020, and six varieties had unknown Years. The second cluster consisted of 41 varieties, accounting for 40.2% of the total varieties, including 2 varieties from 1904-1958, 3 varieties from 1970-1990, 30 varieties from 1990-2020, and 6 varieties with unknown Years. The third cluster encompassed 15 varieties, accounting for 14.7% of the total varieties, including 2 varieties from 1904-1958, 1 variety from 1958-1970, 4 varieties from 1970-1990, 3 varieties from, and 5 varieties with unknown Years.

A variance analysis was performed on all seed phenotypic indicators, which demonstrated that these indicators had statistical significance in all examined varieties. Nonetheless, the seed coat thickness indicator was excluded from the clustering analysis due to its insignificant change. Ten seed phenotypic indicators were standardized using the Z-score method. After standardizing all phenotypic data, we performed Ward clustering, resulting in the classification of 102 varieties into three major clusters based on the squared Euclidean distance (**Figure 7**).

**Figure 8:**
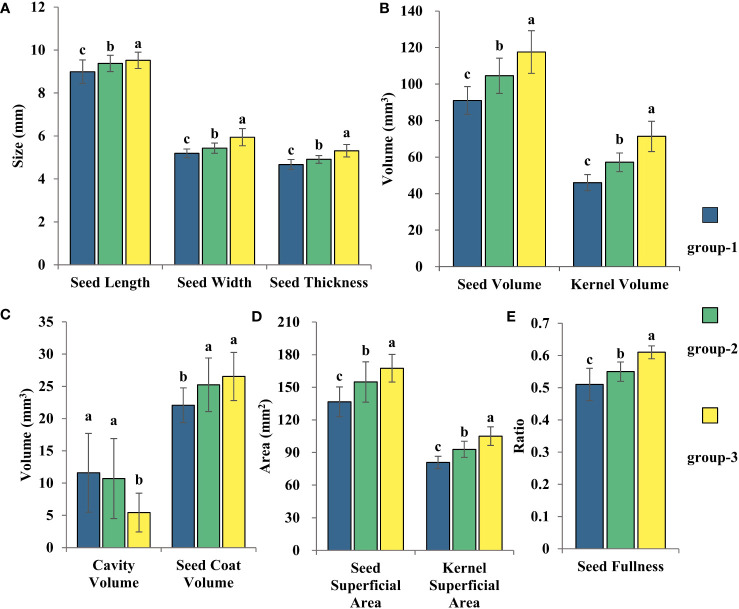
The three clustering results from the statistical analysis of the variations in cotton seed morphology were Seed Length, Width, and Thickness **(A)**, Seed Volume and Seed Kernel Volume **(B)**, Cavity Volume and Seed Coat Volume **(C)**, Seed Surface Area and Seed Kernel Surface Area **(D)**, and Seed Fullness **(E)**. LSD test was used for normal distribution data. Date represents mean ± SE (3 biological replicates, n=46,41 and 15 varieties, respectively), letters above the bars indicate significant differences at the level of P<0.05.

LSD test was conducted on three groups of 102 varieties (**Figure 8**), and the results showed that group-1 was classified as a small seed group with the smallest seed volume and seed kernel volume and the largest cavity volume. The seed size of the group-2 was at a medium level, the cavity volume was slightly lower than that of the group-1, and the seed coat volume was significantly higher than that of the group-1, but there was no significant difference compared with the group-3, so it could be classified as the middle seed group. Group-3 had the largest seed volume and seed kernel volume, but the smallest cavity volume, which is classified as the large seed group.

The 1904-1958 group exhibited an even distribution of varieties among large, medium, and small seed clusters, at 20%, 40%, and 40%, respectively (**Figure S2**). In the 1958-1970 group, 75% of the varieties was distributed in small seed clusters, while the remaining 25% were distributed in large seed clusters (**Figure S2**). The 1970-1990 group showed a roughly similar distribution among three seed clusters, accounting for 36.4%, 27.2%, and 36.4% (**Figure S2**). For the 1990-2020 group, the majority of the varieties were distributed in small and medium seed clusters at 50.8% and 44.4%, respectively (**Figure S2**). Only 4.8% of the varieties was distributed in the large seed cluster (**Figure S2**). Thus, between 1958-2020, the seed morphology of self-bred varieties in China transitioned from first changing from large and small seed groups to three seed groups of large, medium and small, and finally to the change process of middle and small seed groups.

### 3.5 Evaluation of seed morphological structure in different years

**Figure 9:**
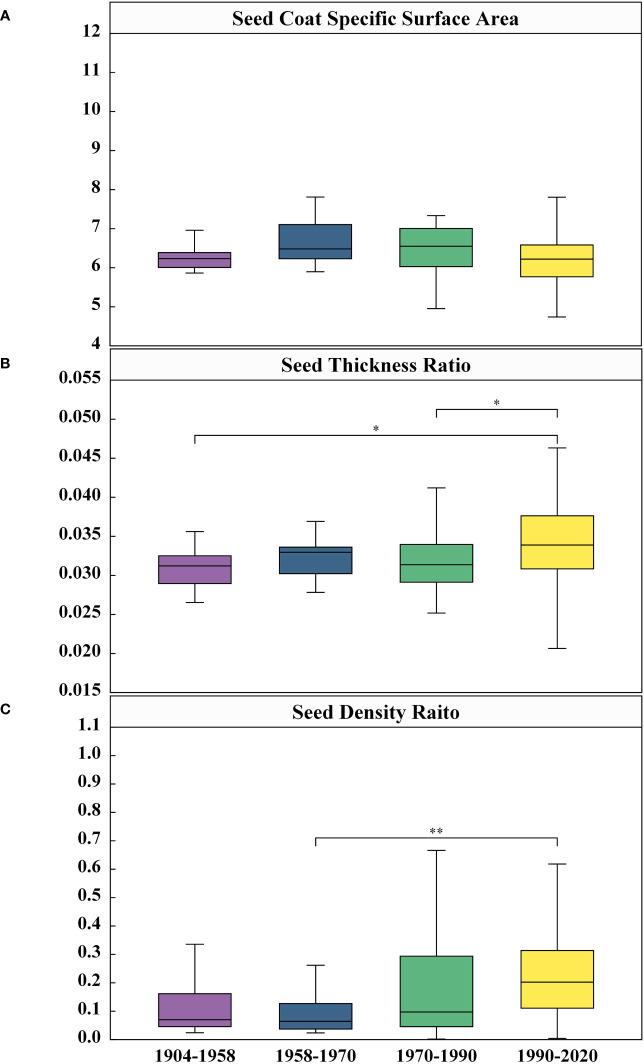
Comparison of differences in Seed Coat Specific Surface Area **(A)**, Seed Coat Thickness Ratio **(B)**, and Seed Density Raito **(C)** among four Years. Given are the means ± SEM. Boxes represent first and third quartile (upper and lower margins), and median (horizontal line). N=306, significance: *<0.05, **<0.01.

In order to facilitate better evaluation of morphological structural differences among different varieties throughout time, this study proposed three specific parameters, namely seed coat specific surface area, seed coat thickness ratio and seed density ratio. The evaluation of seed coat specific surface area among distinct generations of cotton seeds, indicated in **Figure 9A**, illustrates that respective average roughness measurements were 6.31 m^-1^, 6.66 m^-1^, 6.23 m^-1^, and 6.28 m^-1^ for the 1904-1958 through 1990-2020. While the seed coat specific surface area increased initially from 1904-1958 to 1990-2020, it underwent a decreasing trend afterwards, but there was no significant variation in seed coat specific surface area among these generational cohorts. Moreover, implications derived from seed coat thickness ratio analysis (**Figure 9B**) showed a low degree of characterization of seed coat thickness ratio among these distinct years, as seen by the mean value ranging between 0.031 and 0.034 showing an increasing trend — that was, the relative thickness of the seed coat increased. Calculation from **Figure 9C** demonstrated that the mean value of the seed density ratio elevated from 0.15 to 0.23 throughout the 70-year time period. However, for 1958-1970, the seed density ratio was set at 0.10, which is considerably below the recorded values of the remaining years. On the whole, the results of the seed density ratio showed that the seed morphological structure in self-bred varieties in China was becoming more compact. While the cavity was increasing year by year, the seed kernel size was increasing at a faster rate than the cavity. The seed thickness ratio and specific surface area of the seed coat exhibited an upward trend. However, the comparative approach, taking into account the overall morphological structure of the seed, offered a more compelling depiction of the average change in seed coat thickness.

The correlation analysis of the three parameters, presented in **Table 2**, identified a significant association among them. Of these parameters, seed coat specific surface area and seed coat thickness ratio showed the highest correlation coefficient (r = -0.80, P< 0.001), suggesting the likelihood of their representing similar traits. Hence, when assessing seed morphological structure, either of the parameters could be utilized without necessitating the requirement to utilize both.

**Table 2:** Correlation analysis of four seed evaluation indicators.

	Seed Coat Specific Surface Area	Seed Coat Thickness Ratio	Seed Density Ratio
Seed Coat Specific Surface Area	1	-0.80^***^	-0.26^**^
Seed Coat Thickness Ratio		1	0.26^**^
Seed Density Ratio			1

**Represents significant correlation at P<0.01, ***Represents significant correlation at P<0.001.

## 4 Discussion

### 4.1 Analyzing the microscopic phenotype characteristics of seeds

Micro-CT nondestructive imaging technology has been widely used in plant phenomics (Guelpa et al., 2015; Hu et al., 2020; Wu et al., 2021). This paper presented a novel technique for studying the microscopic phenotype of cotton seeds. In most studies, researchers obtained a large number of micro-CT images from single seeds by scanning micro-CT and processing them individually by manual frame selection or commercial software, which took a lot of labor time (Hou et al., 2019; Dong et al., 2020). Therefore, this paper aims to transform the original batch seed kernel micro-CT image processing problem into a single seed kernel segmentation problem. Additionally, the analysis of individual seed kernel components is also transformed into a semantic segmentation problem, focusing on the seed kernel and cavity components that possess the most prominent internal characteristics. The method improved the efficiency of seed treatment and quickly extracted 11 seed structural traits. The micro-CT equipment took 29-30 minutes to scan a single seed, and scans three seeds each time. In contrast to the conventional commercial software utilized for single seed processing, the seed processing technique described in this paper is characterized by its brevity and enhanced efficiency. Consequently, a complete microscopic phenotype technique system for cotton seeds has been established, which provides significant technical support for furthering the exploration of the internal morphological structure of the seeds.

The contrast of the image obtained by micro-CT theoretically depends on the density, thickness and molecular structure of the sample (Lusic and Grinstaf, 2013). Owing to the communication of the seed cavity under a damaged seed coat with the external air (**Figure 3D**), obtaining accurate segmentation and quantification would be impracticable. This makes it difficult to distinguish grayscale pixels. Hence, in this study, only seeds with undamaged seed coats were utilized for accurate segmentation and quantification. Moreover, dehydration in seeds may result in the potential connection of the internal cavity of the seed kernel (**Figure 3E**) with the exterior cavity of the seed kernel, consequently rendering the task of obtaining accurate segmentation and quantification challenging. Furthermore, we also observed the situation of seed kernel breakage (**Figure 3F**), which is comparably infrequent. Given the unclear structure of the kernel, we segmented cotton seeds into three components: seed coat, cavity, and kernel. Here is a study that is similar to our segmentation results. In walnut, researchers only segmented the cavity, seed kernel and shell (Bernard et al., 2020). Conversely, in seeds of monocotyledonous crops, Hou et al. (2019) defined three types of cavities in maize seeds: the embryonic cavity, the endosperm cavity, and the subcutaneous cavity outside the endosperm. However, the seed coat and endosperm of maize seeds are fused together, so the authors treated them as a whole during calculations. Although this paper extracted two phenotypic indicators, seed coat volume and average seed coat thickness, inner and outer seed coat features were not successfully segmented. Further research and exploration are needed to accurately segment seed morphological structures.

Wu et al. (2022) analyzed the correlations of seed traits including weight of 100 seeds, seed length, seed width, seed length-width ratio, seed area, seed perimeter, seed diameter, and sphericity with an automatic seed testing machine and found that these morphological traits are environmentally stable. In the correlation analysis conducted by Wu et al. (2022), the correlation between seed length and seed width was low, while the correlation between the seed width and seed thickness was high, which was consistent with our research results. Nevertheless, there was a strong positive correlation between seed length and thickness, as well as between seed surface area, width, and thickness, which contradicted the findings presented in this paper.

Seed kernel is the best source of seed protein and oil, which relates to seed size (Huang et al., 2022). It means that a larger seed kernel size may produce more nutrients. Our study found a positive correlation between seed kernel size and seed volume, which is consistent with previous research results (Bernard et al., 2020). In addition, the condition of the kernel is an important indicator of seed quality, and larger cotton seed kernels contribute to seedling growth (Ahmed et al., 2020). Clearly, seed kernel size is mainly influenced by seed coat and size; that is, the larger the seed, the larger the seed kernel. Micro-CT scanning of the seed’s internal shape and structural characteristics is a reliable method to predict cotton growth.

In this study, the variation coefficient of cavity volume was too high. Correlation analysis of 102 cultivars indicated a positive correlation between seed coat volume and cavity volume, suggesting that the formation of cotton seed cavities may be related to the morphological structure of the seed coat. However, it is unclear whether this structural feature varies among cultivars, and further exploration is necessary to determine the reasons for the high variability coefficient.

Generally, the variability coefficient of average seed coat thickness was small across cultivars, and average seed coat thickness had no significant correlation with most phenotypes except for those related to seed coat volume. This is consistent with the findings of Bernard et al. (2020). Therefore, there may be unidentified factors that affect the average seed coat thickness.

### 4.2 Differences and evaluation of seeds in different years

Understanding the changing trend of seed size is critical for germplasm enhancement. Researching the evolution law of germplasm contributes significantly to the growth and regulation of cotton seeds and organs, and has vital implications in setting breeding objectives, selecting parent materials, and offspring (Huang et al., 2022).

The application of computed tomography scanning technology has revealed the correlation between small cavities in soybeans and their oil content. More small cavities are present in modern soybeans compared to ancient soybeans, suggesting a gradual upsurge in soybean oil demand (Zong et al., 2017). It indicates that micro-CT has significant potential to explore the crop domestication process. Our study classified 83 cotton varieties into four distinct periods and uncovered various alterations in seed morphological structure throughout the course of cotton development in China. Our research exhibited that there were notable variations in seed coats, seed kernels, and cavities amongst cotton seeds from different periods. However, the average seed coat thickness did not demonstrate a noticeable trend. There was a growing trend in the seed coat, kernel, and cavity sizes of self-bred varieties in China. This suggested that the focus of breeding bred varieties in China is on increasing seed and kernel sizes, with less emphasis on the average seed coat thickness.

This paper presents three innovative indicators to assess the morphological structure of the seeds. Our objective is to improve the understanding of the relationship between seed size, seed coat thickness, and internal morphological structure throughout the breeding process. Among them, the seed coat specific surface area of domestic self-bred varieties in China were showing a decreasing trend. This may be because with a larger specific surface area, water is absorbed more quickly for seed, and the rapid absorption of water can damage seed cells and affect the cleanliness of seedlings (Main et al., 2014). These indicators could help develop better breeding plans and strategies. The indicators proposed in this paper also reflected the differences in seed relative thickness and internal morphological structure compactness between China’s self-bred modern varieties and foreign-introduced varieties. As modern varieties replaced the old, the gap between these indicators and foreign varieties gradually narrows.

Unlike the previous approach of only judging the seed size based on one-dimensional data, our study introduced 3D phenotypic indicators in addition to traditional one-dimensional data for seed classification, as opposed to solely assessing seed size. Our study revealed that between 1958-2020, seed morphology in China transitioned from first changing from large and small seed groups to three seed groups of large, medium and small, and finally to the change process of middle and small seed groups. The shift in seed size may be due to two primary factors. First, the medium seed group exhibits a more significant response to nitrogen use than both the larger and smaller seed groups. Large seed varieties exhibit longer fibers, greater fiber strength, improved uniformity in fiber length, and smaller particle sizes compared to small seed varieties (Main et al., 2014). Second, in terms of nutrient effects, small seeds exhibit faster germination and emergence compared to large seeds. Crops with larger seeds necessitate greater nutrient accumulation for germination and emergence, potentially impacting seed health and uniform emergence rates (Wang et al., 2008; Vidak et al., 2022). Consequently, breeders tend to favor smaller seeds in the cultivation process to achieve optimal growth conditions.

### 4.3 Seed morphological structure and germination prediction

Researchers often use micro-CT to analyze the structural characteristics of seeds and predict their germination potential. Through micro-CT images of chili peppers and germination tests, Ahmed et al. (2020) found that the shape and length of the embryonic root correlated with the germination quality of seeds. They believed that seeds with compact internal morphological structures (low air cavity ratio) and appropriate kernel shapes were able to germinate better. Additional investigation is required to further explore the correlation between the volume and proportion of the morphological structure of cotton seeds and the process of cotton seed germination. However, some studies have shown that certain doses of X-rays may kill or cause mutations in seeds, leading to abnormal kernel morphological structures such as degeneration, folding, lateral bending, and fracture (Gargiulo et al., 2020). Therefore, when exploring the relationship between seed morphology and germination, attention should be paid to the effects of X-ray radiation dose and exposure time on seed germination.

## 5 Conclusions

Using micro-CT scanning, this study conducted quantitative and comparative analyses of the morphological structure of 102 cotton seeds through 3D reconstruction and image segmentation. A non-destructive high-throughput analysis method was established to accurately identify the linear size, volume, and other indicators of seeds, as well as quantify phenotype indicators such as seed surface area, seed kernel volume, seed kernel surface area, cavity volume, seed coat volume, and average seed coat thickness. The study demonstrated a positive correlation between seed kernel size and seed size, while seed cavity size and average seed thickness were less influenced by other morphological indicators. During the period between 1904 and 2020, the overall trend in the physical morphological structure of cotton seeds in China decreased. However, for locally-bred cotton varieties (1958-2020), the size of the physical morphological structure of the seed increased, then decreased, demonstrating an overall increasing trend in size. Cluster analysis results showed that the seed type of China’s independently bred cotton varieties underwent a transformation from large and small seed groups to large, medium, and small seed groups, and then to medium and small seed groups. The study proposes three seed morphological structure evaluation indicators, indicating that with the replacement of varieties, the specific surface area of the seed increases, the relative thickness of the seed coat increases, and the internal morphological structure of the seed becomes denser. Overall, these findings demonstrate that the morphological evolution history of cotton seeds in China provides important theoretical support for cotton variety breeding and seed quality evaluation.

## Data availability statement

The raw data supporting the conclusions of this article will be made available by the authors, without undue reservation.

## Author contributions

XG conceived and designed the experiments. YL, DL, and MG carried out the experiment and collected image data. YL and GH performed data processing and analysis, and drafted the manuscript. XG and YoZ is the supervisor of YL and participated in the guiding the writing of this manuscript. XL, SG, and YiZ provided the technical guidance and editing support. All authors contributed to the article and approved the submitted version.
