# Supplementary material for: Research on the evolutionary history of the morphological structure of cotton seeds: a new perspective based on high-resolution micro-CT technology
Source: Front Plant Sci. 2023 Oct 13;14:1219476. doi: 10.3389/fpls.2023.1219476 (PMC10613036; doi:10.3389/fpls.2023.1219476)
Supplement: Supplementary file 1 [file DataSheet_1.docx]

Supplementary Material

# Supplementary Figures and Tables

## Supplementary Figures


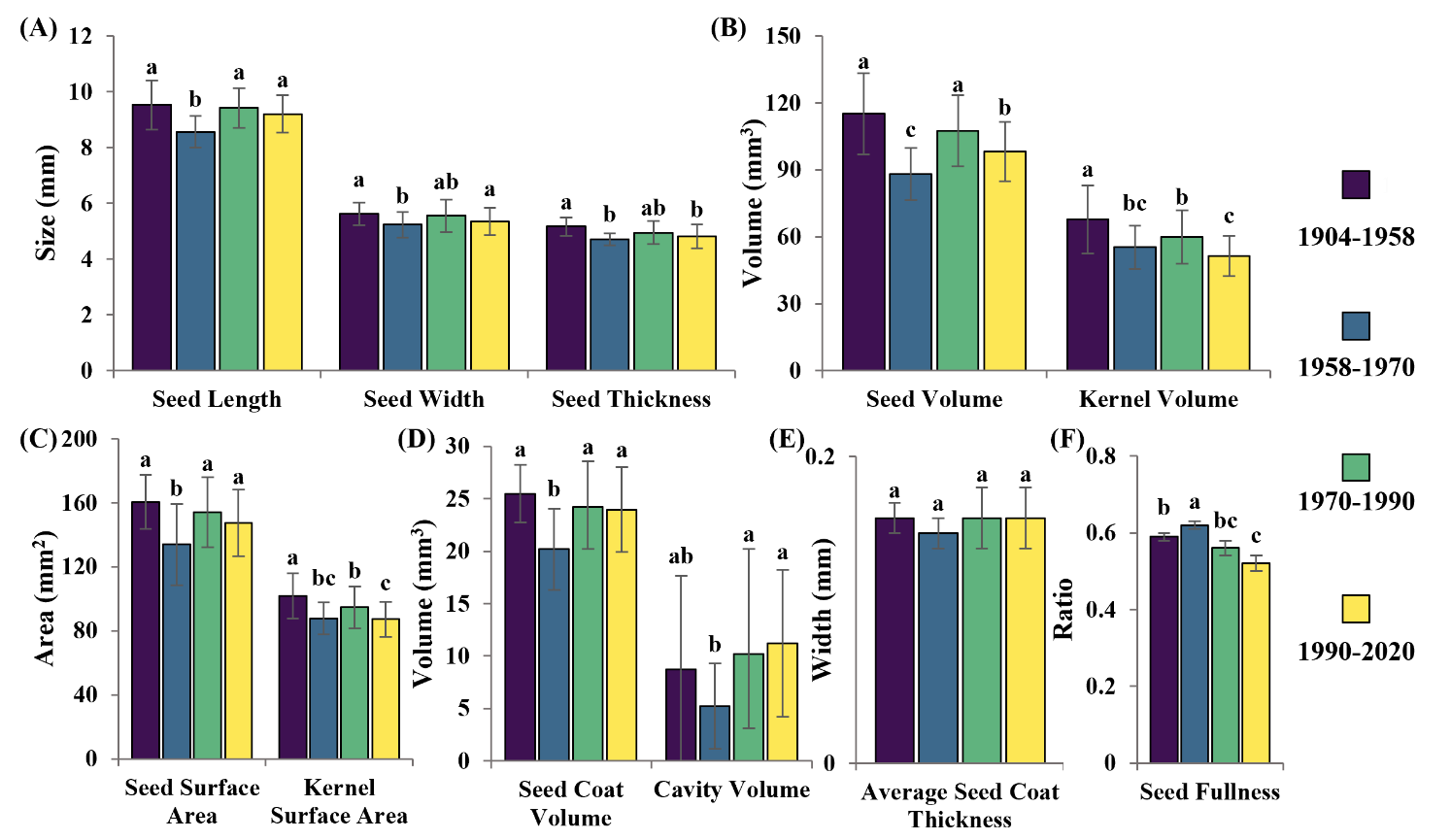


**Figure S1.** Cotton seeds in four years: seed length, width, and thickness (A), seed volume, kernel volume (B), seed surface area, kernel surface area (C), cavity volume, seed coat volume (D), and statistical analysis of variations in average seed coat thickness (E) and seed fullness (F). LSD test was used for normal distribution data. Date represent mean±SE (3 biological replicates, n=15,12,33 and 189 varieties, respectively), letters above the bars indicate significant differences at the level of P<0.05.


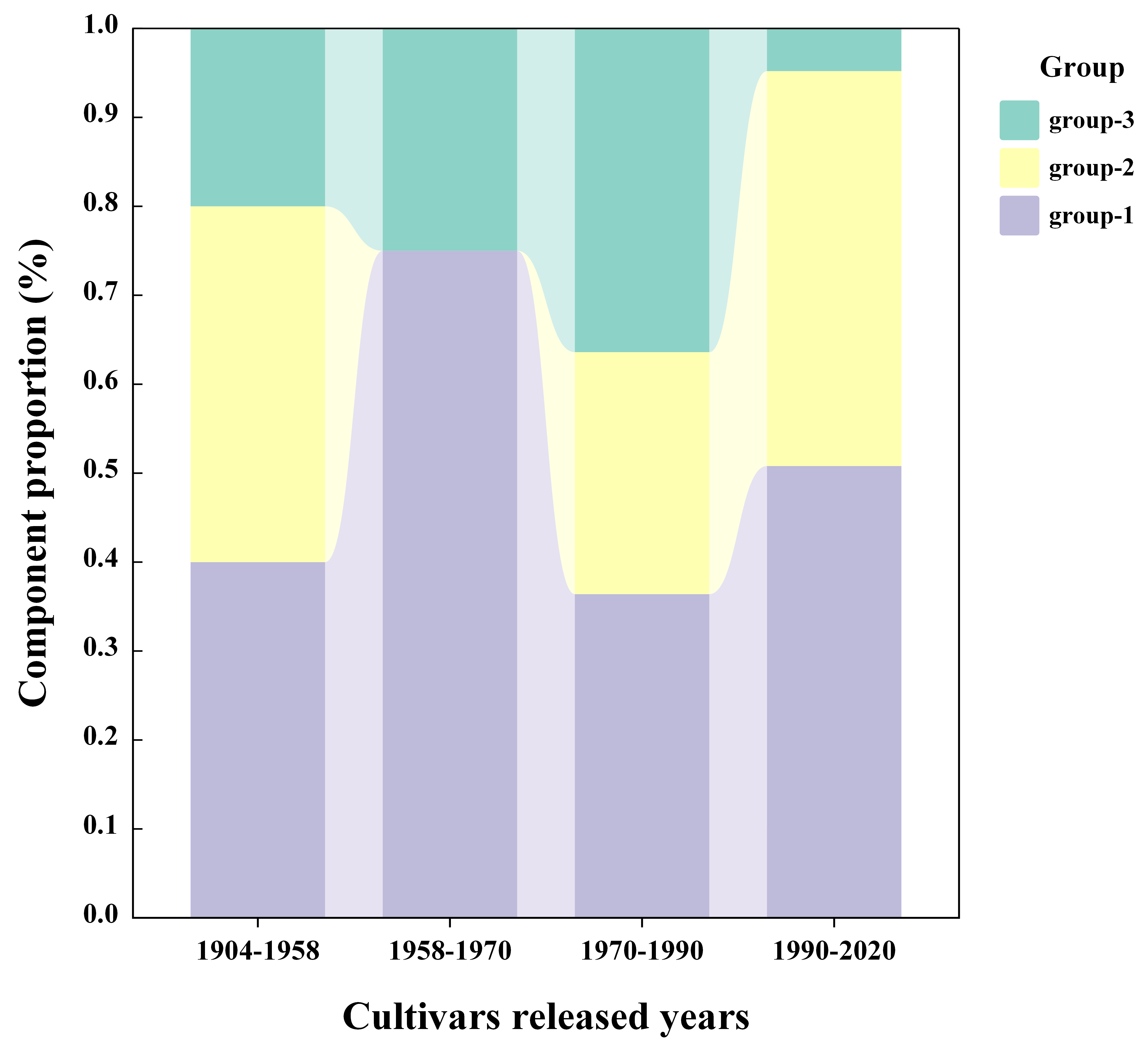


**Figure S2.** The proportion of components of different types of seeds in the varieties of four years. n= 15,12,33,189 varieties, respectively. Among them, group-1 is small seed group, group-2 is a medium seed group and group-3 is a large seed group.

## Supplementary Tables

| **Table S1. Cotton Resources information used in Micro-CT study** | | | | | | | |
| --- | --- | --- | --- | --- | --- | --- | --- |
|  | Variety | Abbreviation | Classification |  | Variety | Abbreviation | Classification |
| 1 | 86-1 | 86-1 | 3 | 52 | Xinluzao21Hao | XLZ21H | 3 |
| 2 | Baimian1Hao | BM1H | 4 | 53 | Xinluzao24Hao | XLZ24H | 4 |
| 3 | Bomian1Hao | BoM1H | unknown | 54 | Xinluzao25Hao | XLZ25H | 4 |
| 4 | Cang198 | C198 | 4 | 55 | Xinluzao26Hao | XLZ26H | unknown |
| 5 | Daizimian20 | DZM20 | 1 | 56 | Xinluzao27Hao | XLZ27H | 4 |
| 6 | DPmian | DPM | unknown | 57 | Xinluzao31Hao | XLZ31H | 4 |
| 7 | Emian9Hao | EM9 | unknown | 58 | Xinluzao36Hao | XLZ36H | unknown |
| 8 | Ganmian4Hao | GM4 | unknown | 59 | Xinluzao41Hao | XLZ41H | 4 |
| 9 | Guangyedaizimian | GYDZM | 1 | 60 | Xinluzao45Hao | XLZ45H | 4 |
| 10 | Guannong1Hao | GN1 | 3 | 61 | Xinluzao46Hao | XLZ46H | 4 |
| 11 | Guoxin2Hao | GX2 | unknown | 62 | Xinluzao48Hao | XLZ48H | 4 |
| 12 | Guoxin3Hao | GX3 | 4 | 63 | Xinluzao50Hao | XLZ50H | 4 |
| 13 | Guoxin9Hao | GX9 | 4 | 64 | Xinluzao53Hao | XLZ53H | 4 |
| 14 | Hai92-4 | H92-4 | unknown | 65 | Xinluzao6Hao | XLZ6H | 4 |
| 15 | Handan9842 | HD8942 | unknown | 66 | Xinluzhong11Hao | XLZ11H | unknown |
| 16 | Ji2658 | J2658 | 4 | 67 | Xinluzhong26Hao | XLZ26H | 4 |
| 17 | Ji589 | J589 | 4 | 68 | Xuzhou1818 | XZ1818 | 2 |
| 18 | Ji668 | J668 | 4 | 69 | Zhong21371 | Z21371 | unknown |
| 19 | Jifeng914 | JF914 | 4 | 70 | Zhong375 | Z375 | 3 |
| 20 | Jimian646 | JM646 | 4 | 71 | Zhong425 | Z425 | 4 |
| 21 | Jmian958 | JM958 | 4 | 72 | Zhong50 | Z50 | 4 |
| 22 | Jinkemian11Hao | JKM11H | 4 | 73 | Zhongmian100 | ZM100 | 4 |
| 23 | Junhai1Hao | JH1H | 3 | 74 | Zhongmiansuo10Hao | ZMS10H | 3 |
| 24 | Junmian1Hao | JM1H | 3 | 75 | Zhongmiansuo12Hao | ZMS12H | 3 |
| 25 | kekangmian1Hao | KKM1H | 4 | 76 | Zhongmiansuo2Hao | ZMS2H | 2 |
| 26 | Keke1543 | KK1543 | 1 | 77 | Zhongmiansuo3Hao | ZMS3H | 2 |
| 27 | Lumian1Hao | LM1H | 3 | 78 | Zhongmiansuo41 | ZMS41 | 4 |
| 28 | Lumian28 | LMY28 | 4 | 79 | Zhongmiansuo42 | ZMS43 | 4 |
| 29 | Lumianyan37 | LMY37 | 4 | 80 | Zhongmiansuo45 | ZMS45 | 4 |
| 30 | Nandanbadidahua | NDBDDH | unknown | 81 | Zhongmiansuo5Hao | ZMS5H | 3 |
| 31 | Jinongda36Hao | ND36 | 4 | 82 | Zhongmiansuo60 | ZMS60 | 4 |
| 32 | Nongdamian23Hao | NDM23H | 4 | 83 | Zhongmiansuo79 | ZMS79 | 4 |
| 33 | Nongda601 | ND601 | 4 | 84 | ZhongR2007 | ZR2007 | 4 |
| 34 | Nuohuamian1Hao | NHM1H | 4 | 85 | Haidaomian3-79 | HDM3-79 | unknown |
| 35 | Shannongshengmian1Hao | SNSM1H | 4 | 86 | Xinshi17 | XS17 | 4 |
| 36 | Shikang126 | SK126 | 4 | 87 | Ekangmian9Hao | EKM9H | 4 |
| 37 | Shiyuan321 | SY321 | 4 | 88 | Ezamian3Hao | EZM3H | 4 |
| 38 | Shuangjia321 | SJ321 | unknown | 89 | Ji228 | J228 | 4 |
| 39 | Shuozamian2Hao | SZM2H | 4 | 90 | Lumianyan40 | LMY40 | 4 |
| 40 | Sikang1Hao | SK1H | 4 | 91 | Lumianyan17Hao | LMY17 | 4 |
| 41 | Simian3Hao | SM3H | 4 | 92 | Nongda7Hao | ND7H | 4 |
| 42 | Sizimian2B | SZM2B | 1 | 93 | Sukangmian1Hao | SKM1H | 4 |
| 43 | Sizimian4B | SZM4B | 1 | 94 | Sumian22Hao | SM22H | 4 |
| 44 | Sumain12 | SM12 | 4 | 95 | Xipu6 | XP6 | 4 |
| 45 | Sumian15 | SM15 | 4 | 96 | Xingtai6871 | XT6871 | 2 |
| 46 | Tongjian1Hao | TJ1H | unknown | 97 | Yumian11Hao | YM11H | 4 |
| 47 | Wanfeng203 | WF203 | 4 | 98 | Pima90-53 | PIMA90-53 | unknown |
| 48 | Xinhai20 | XH20 | unknown | 99 | Tu76-94 | T76-94 | unknown |
| 49 | Xinlumian1Hao | XLM1H | 4 | 100 | Xinluzao33Hao | XLZ33H | 4 |
| 50 | Xinluzao11Hao | XLZ11H | 4 | 101 | Jinmian13Hao | JM13H | 4 |
| 51 | Xinluzao1Hao | XLZ1H | 3 | 102 | Zhong9806 | Z9806 | unknown |

| **Table S2 Cotton seed three-dimensional morphological traits measured by micro-CT images** | | |
| --- | --- | --- |
| External characteristics | Description | Unit |
| Seed Volume | Volume of cotton seed, including the sum of kernel volume, cavity volume, and seed coat volume. | mm³ |
| Seed Surface Area | Surface area of the cotton seed, specifically referring to the surface area of the seed coat. | mm^2^ |
| Seed Length | The longest possible straight-line from the cotton seed's base to its top. | mm |
| Seed Width | The greatest distance in a straight-line from the cotton seed's largest cross section. | mm |
| Seed Thickness | The cotton seed's minimal straight-line distance from its largest cross section. | mm |
| Seed Coat Volume | The volume of cotton seed coat. | mm³ |
| Average Seed Coat Thickness | Average cotton seed coat thickness, defined as the ratio of seed coat volume to surface area. | mm |
| Seed Kernel Volume | The volume of cotton seed kernel. | mm³ |
| Seed Kernel Surface Area | The surface area of cotton seed kernel. | mm^2^ |
| Seed Fullness | The ratio of kernel volume to the whole cotton seed volume. | % |
| Cavity Volume | The volume of the cavity between the cotton seed coat and kernel. | mm³ |
